# HIV infection is associated with higher levels of monocyte chemoattractant protein-1 and eotaxin among people with recent hepatitis C virus infection

**DOI:** 10.1186/s12879-016-1567-2

**Published:** 2016-06-01

**Authors:** François M. J. Lamoury, Behzad Hajarizadeh, Elizabeth Keoshkerian, Jordan J. Feld, Janaki Amin, Suzy Teutsch, Gail V. Matthews, Margaret Hellard, Gregory J. Dore, Andrew R. Lloyd, Tanya L. Applegate, Jason Grebely, on behalf of the ATAHC Study Group

**Affiliations:** The Kirby Institute, UNSW Australia, Sydney, Australia; Inflammation and Infection Research Centre, School of Medical Sciences, UNSW Australia, Sydney, Australia; Toronto Centre for Liver Disease, McLaughlin-Rotman Centre for Global Health, University of Toronto, Toronto, Canada; HIV/Immunology/Infectious Diseases Clinical Services Unit, St Vincent’s Hospital, Sydney, Australia; Burnet Institute, Melbourne, Australia; Viral Hepatitis Clinical Research Program, The Kirby Institute, UNSW Australia, Sydney, NSW 2010 Australia

**Keywords:** HCV, Cytokines, Chemokines, Co-infection, Acute infection

## Abstract

**Background:**

Human immunodeficiency virus (HIV) infection leads to more rapid progression of hepatitis C virus (HCV)-related liver fibrosis, which could be linked to differences in the severity of liver inflammation among HIV/HCV co-infected individuals compared to HCV mono-infected individuals.

This study assessed the association of HIV co-infection with pro-inflammatory and pro-fibrogenic cytokines and chemokines during recent HCV infection.

**Methods:**

Participants from the ATAHC study, a prospective cohort of recent HCV infection, with detectable HCV RNA at the time of acute HCV detection were included. Concentrations of 27 plasma cytokines and chemokines were measured by multiplex immunoassays and compared between those with, and without, HIV co-infection.

**Results:**

Out of 117 individuals with recent HCV infection included in analysis, 73 had HCV mono-infection and 44 had HIV/HCV co-infection. Individuals with HIV/HCV co-infection had significantly higher mean levels of eotaxin (1.79 vs. 1.62 log pg/mL; *P* < 0.001), monocyte chemotactic protein 1 (MCP-1; 2.10 vs. 1.98 log pg/mL; *P* < 0.001), and interferon-gamma inducible protein-10 (IP-10; 3.11 vs. 2.98 log pg/mL; *P* = 0.013). Linear regression analyses adjusting for age, alanine transaminase (ALT), HCV RNA levels, and assay run, higher eotaxin levels were independently associated with HIV/HCV co-infection (adjusted β: 0.12; 95%CI: 0.01, 0.24; *P* = 0.039). Higher MCP-1 levels were also independently associated with HIV/HCV co-infection in adjusted analysis (adjusted β: 0.11; 95%CI: 0.03, 0.18; *P* = 0.009).

**Conclusions:**

During recent HCV, those with HIV/HCV co-infection had a stronger pro-fibrogenic mediator profile compared to those with HCV mono-infection. These findings may provide a potential explanation for accelerated liver fibrosis in HIV/HCV co-infection.

**Trial registration:**

Australian Trial in Acute Hepatitis C (ATAHC) study was registered with ClinicalTrials.gov registry on September 11, 2005. NCT00192569.

**Electronic supplementary material:**

The online version of this article (doi:10.1186/s12879-016-1567-2) contains supplementary material, which is available to authorized users.

## Background

Among people with chronic hepatitis C virus (HCV) infection, co-infection with human immunodeficiency virus (HIV) leads to accelerated liver fibrosis progression [1–5]. Several mechanisms have been suggested, including increased pro-fibrogenic cytokine expression and secretion, enhanced oxidative stress, increased hepatocyte apoptosis, and immunosuppression [1, 6–8], although there is no clear consensus.

One potential explanation for more rapid fibrosis progression among HIV/HCV co-infected individuals compared to HCV mono-infected individuals could be differences in the severity of hepatic inflammation, leading to fibrosis. In the setting of chronic HCV infection, it has been demonstrated that individuals with HIV/HCV co-infection have higher levels of a number of pro-inflammatory cytokines and chemokines [9, 10]. Further, it has been shown that intrahepatic mRNA levels of inflammatory cytokines are higher among people with HIV/HCV co-infection as compared to HCV mono-infection [11]. In the setting of acute HCV infection, it has previously been demonstrated that higher levels of interferon-gamma inducible protein-10 (IP-10) levels are observed among people with HIV, compared to those without HIV infection [12]. However, there are little data evaluating other cytokine and chemokine levels in people with, and without, HIV co-infection with recent HCV infection.

The Australian Trial in Acute Hepatitis C (ATAHC) was a multicentre, prospective cohort study of the natural history and treatment of recent (acute and early chronic) HCV infection [13]. The aim of this study was to compare cytokine and chemokine levels in people with, and without, HIV co-infection who acquired HCV infection.

## Methods

### Study participants

In ATAHC, acute or early chronic HCV infection was defined by an initial positive anti-HCV antibody test within 6 months of enrolment and either 1) a negative anti-HCV antibody test within 2 years prior to the initial positive anti-HCV antibody test or 2) acute clinical hepatitis within 12 months before the initial positive anti-HCV antibody result. Acute clinical infection was defined by symptomatic seroconversion illness or peak alanine transaminase (ALT) level greater than 400 IU/mL at or before the time of HCV diagnosis. In the current study, ATAHC participants with available plasma samples and HCV RNA detected at the time of acute HCV detection (screening visit) were included. Cytokines and chemokines were measured in screening visit plasma samples using a multiplex assay (see below).

### Measurement of plasma cytokines and chemokines

Three human cytokine multiplex bead array assay kits utilizing technology licensed by Luminex (Bio-Rad, Gladesville, Australia) measured the following cytokines and chemokines: interleukin-1b (IL-1b), IL-4, IL-6, IL-10, IL-17A, IL-17F, IL-21, IL-22, IL-23, IL-25, IL-31, IL-33, interferon-gamma (IFN-g), soluble CD40 ligand (sCD40L), tumor necrosis factor alpha (TNF-a), (with Bio-Plex human TH17 15-plex), IL-2, IL-8, eotaxin-1 (or CCL11), IFN-g, interferon-gamma inducible protein-10 (IP-10, or CXCL10), monocyte chemotactic protein 1 (MCP-1, or CCL2), macrophages inflammatory proteins 1 alpha (MIP-1a, or CCL3), MIP-1ß (or CCL4), RANTES (regulated upon activation normal T-cell expressed, and presumably secreted; or CCL5) (with Bio-Plex human cytokine Group I 9-plex) and IL-18, TNF-ß, TNF-related apoptosis-inducing ligand (TRAIL, or TNFSF10) (with Bio-Plex human cytokine Group II 3-plex).

The protocol was performed as per the manufacturer’s instructions and previously described [14, 15]. Samples were centrifuged at 10,000 x g for 10 min at 4 °C to remove platelets and precipitates, after which the supernatants were diluted four times with assay diluents. The assay was performed using all the assay components provided in a 96-well filter plate. The raw data was analysed using the Bio-Plex Manager software, v6.1 (Bio-Rad) [16].

### Statistical analysis

Log_10_ transformed values of cytokines levels (log_10_ pg/mL) were used in analysis given the distribution of the actual values were not normal. The mean of plasma cytokine levels were compared between individuals with and without HIV co-infection (Student *T* test). If the plasma cytokine level was below the level of detection, the midpoint between zero and the lowest level of reliable detection was imputed (Additional file 1: Table S1). Plasma levels of four cytokines (IL-23, IFN-g, TNF-b, and MIP-1a) were undetectable in ≥20 % of individuals, for which proportion of individuals with undetectable cytokine level was compared between individuals with, and without, HIV co-infection (Chi-squared test).

Overall, 27 comparisons of plasma cytokines levels were conducted between two groups of individuals with, and without, HIV co-infection. Whether adjustments are needed for multiple comparisons is a matter of controversy given that multiple comparison testing might inflate type 1 error while adjustments for multiple comparisons inflate type 2 error [17–20]. To account for multiple comparisons, a moderately conservative significance level (*alpha* = 0.01) was used. In a sensitivity analysis, using Bonferroni correction for multiple comparisons, an *alpha* = 0.05/27 = 0.002 was also used, but the findings were similar.

Two cytokines (i.e., MCP-1 and eotaxin) which had significantly different means between individuals with, and without, HIV co-infection were included in linear regression analysis. Linear regression models were fitted to assess the association of HIV co-infection with each plasma cytokine levels (log pg/mL). In the adjusted models, the association of these two cytokines and HIV co-infection was adjusted for potential confounders including age, ALT levels, and HCV RNA level at the time of acute HCV detection. Potential confounders were the variables associated with either HIV status or plasma cytokine levels in this study. To account for potential unmeasured confounders introduced by different assay runs and sample set, the models were also adjusted for assay set. All analyses were performed using Stata v12.0 (College Station, TX, United States).

## Results

### Participant characteristics

A total of 117 individuals with recent HCV infection were included in the study, including 44 (38 %) with and 73 without (62 %) HIV co-infection. Background characteristics of the study population are summarized in Table 1. All individuals with HIV co-infection were male. Compared to individuals without HIV, those with HIV co-infection were significantly older, had a higher ALT level and had a greater proportion with high HCV RNA levels (≥400,000 IU/mL) at the time of recent HCV detection (Table 1). Two participants had HBV co-infection (positive HBsAg), both with no HIV co-infection.

**Table 1:** Baseline characteristics of ATAHC participants with detectable HCV RNA at the time of acute HCV detection, stratified by HIV status

	Total *(n = 117)*	No HIV co-infection *(n = 73)*	HIV co-infection *(n = 44)*	*P*
	n (%) ^a^	n (%) ^a^	n (%) ^a^	
Sex				<0.001
Male	88 (75)	44 (60)	44 (100)	
Female	29 (25)	29 (40)	0 (0)	
Mean age, year (SD)	34 (10)	31 (9)	41 (8)	<0.001
Symptomatic acute HCV				0.825
No	68 (58)	43 (59)	25 (57)	
Yes	49 (42)	30 (41)	19 (43)	
ALT level at acute HCV detection, log_10_ IU/L	2.2 (0.5)	2.1 (0.5)	2.4 (0.5)	0.006
Estimated duration of infection at acute HCV detection				0.236
<26 weeks	69 (59)	40 (55)	29 (66)	
≥26 weeks	48 (41)	33 (45)	15 (34)	
*Interferon lambda 4* genotype (rs12979860)				0.185
TT/CT	56 (48)	37 (51)	19 (43)	
CC	59 (50)	37 (49)	23 (53)	
Unknown	2 (2)	0 (0)	2 (4)	
HCV RNA level at acute HCV detection				0.031
<400,000 IU/mL	78 (67)	54 (74)	24 (55)	
≥400,000 IU/mL	39 (33)	19 (26)	20 (45)	
HCV genotype				0.682
Genotype 1	67 (57)	41 (56)	26 (59)	
Genotype 3	40 (34)	27 (37)	13 (29)	
Other genotypes^b^	8 (7)	4 (5)	4 (9)	
Unknown genotype	2 (2)	1 (1)	1 (2)	

Abbreviation: *SD* standard deviation

^a^Percentages indicate column percentages

^b^Including genotype 2 (*n* = 6), genotype 4 (*n* = 1) and mixed genotype (*n* = 1)

Among 44 individuals with HIV co-infection, 24 (55 %) had HIV viral suppression (≤50 IU/ml) at the time of recent HCV detection. Among those with no HIV viral suppression, the median HIV RNA levels at the time of recent HCV detection was 4400 IU/mL (inter quartile range: 400, 33467). CD4 count at the time of acute HCV detection was ≥500 cells/μL in 28 (64 %) individuals, and 200–499 cells/μL in 14 (32 %) individuals (not available in two individuals). Thirty-three individuals (75 %) were on antiretroviral therapy at the time of acute HCV detection, among whom, 18 (55 %) had HIV viral suppression.

During the follow-up, ten individuals (9 %) cleared HCV spontaneously, while 107 individuals (91 %) either progressed to chronic infection, or received treatment.

### Distribution of cytokines levels by HIV status

**Fig. 1:**
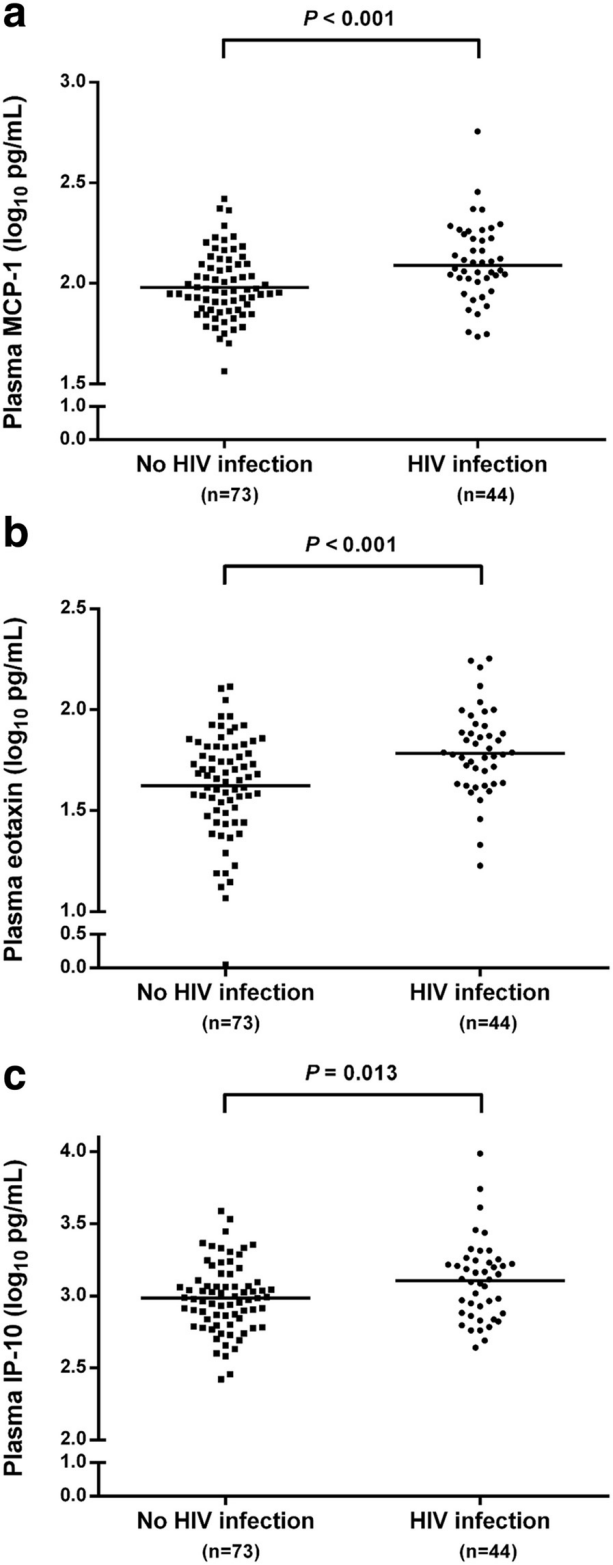
Distribution of MCP-1 (**a**), eotaxin (**b**) and IP-10 (**c**) plasma levels among ATAHC participants with detected HCV RNA at acute HCV detection, by HIV status. *Horizontal lines* represent the mean

The distribution of cytokine levels in individuals with, and without, HIV co-infection is summarized in Table 2. Individuals with HIV co-infection had significantly higher mean plasma levels of MCP-1 and eotaxin compared to those without HIV (*P* < 0.001; Fig. 1a and b). IP-10 also had higher mean plasma levels in those with HIV co-infection compared to those without HIV co-infection (*P* = 0.013; Fig. 1c).

**Table 2:** Plasma cytokine and chemokine levels among ATAHC participants with detectable HCV RNA at the time of acute HCV detection, stratified by HIV

Cytokine	No HIV co-infection *(n = 73)*	HIV co-infection *(n = 44)*	*P*
IL-1 beta^a^	0.77 (0.77)	0.56 (0.59)	0.197
IL-2^a^	1.11 (0.12)	1.09 (0.13)	0.427
IL-4^a^	2.16 (0.25)	2.13 (0.28)	0.589
IL-6^a^	1.60 (0.63)	1.60 (0.64)	0.977
IL-8^a^	1.71 (0.54)	1.71 (0.48)	0.994
IL-10^a^	2.16 (0.66)	1.94 (0.72)	0.100
IL-17A^a^	2.05 (0.53)	1.89 (0.70)	0.212
IL-17F^a^	2.34 (0.49)	2.17 (0.56)	0.088
IL-18^a^	2.44 (0.29)	2.52 (0.25)	0.171
IL-21^a^	3.13 (0.55)	3.07 (0.56)	0.566
IL-22^a^	1.73 (0.68)	1.81 (0.53)	0.458
IL-23^b^	16 (22)	14 (32)	0.252
IL-25^a^	1.46 (0.72)	1.32 (0.94)	0.415
IL-31^a^	2.58 (0.51)	2.53 (0.61)	0.641
IL-33^a^	3.49 (0.26)	3.41 (0.29)	0.245
IFN-gamma^a^	2.62 (0.36)	2.68 (0.39)	0.417
IFN-gamma2^b^	57 (79)	38 (86)	0.329
TNF-alpha^a^	1.30 (0.47)	1.27 (0.47)	0.776
TNF-beta^b^	68 (93)	43 (98)	0.407
TRAIL^a^	2.07 (0.18)	2.12 (0.21)	0.119
sCD40L^a^	2.89 (0.26)	2.90 (0.25)	0.865
CXCL10 (IP-10) ^a^	2.98 (0.24)	3.11 (0.28)	0.013
CCL2 (MCP-1) ^a^	1.98 (0.17)	2.10 (0.19)	<0.001
CCL3 (MIP-1a) ^b^	36 (49)	16 (36)	0.172
CCL4 (MIP-1b) ^a^	2.62 (0.24)	2.70 (0.32)	0.158
CCL5 (RANTES) ^a^	3.78 (1.66)	3.94 (1.56)	0.606
CCL11 (eotaxin) ^a^	1.62 (0.30)	1.79 (0.21)	<0.001

^a^ Presented as mean plasma level (standard deviation); log_10_ pg/mL

^b^ Presented as the number with undetectable plasma level (%)

In unadjusted linear regression analysis, HIV co-infection was significantly associated with higher plasma MCP-1 levels [log pg/mL; β (estimated mean difference): 0.13; 95 % CI: 0.06, 0.19; Table 3]. In the adjusted model, HIV co-infection remained highly significantly associated with higher MCP-1 levels (log pg/mL; adjusted β: 0.11; 95 % CI: 0.03, 0.18; Table 3).

**Table 3:** Unadjusted and adjusted models assessing the association of HIV co-infection with plasma MCP-1 levels (log_10_ pg/mL) in ATAHC

	Unadjusted model		Adjusted model^a^	
	Estimated mean difference^b^ (95 % CI)	*P*	Estimated mean difference^b^ (95 % CI)	*P*
HIV co-infection				
Negative	Reference		Reference	
Positive	0.13 (0.06, 0.19)	<0.001	0.11 (0.03, 0.18)	0.009
Age, 10 years	0.04 (0.00, 0.07)	0.024	0.01 (−0.03, 0.05)	0.575
Symptomatic acute HCV				
No	Reference			
Yes	0.06 (−0.01, 0.13)	0.080		
ALT level, log IU/L	0.07 (0.00, 0.14)	0.049	0.02 (−0.05, 0.10)	0.502
Estimated duration of infection				
<26 weeks	Reference			
≥26 weeks	−0.05 (−0.12, 0.02)	0.196		
*Interferon lambda rs12979860* genotype				
TT/CT	Reference			
CC	0.01 (−0.06, 0.08)	0.809		
HCV RNA level				
<400,000 IU/mL	Reference		Reference	
≥400,000 IU/mL	0.05 (−0.02, 0.12)	0.181	0.02 (−0.06, 0.10)	0.623
HCV genotype^c^				
Genotype 1	Reference			
Genotype 3	0.00 (−0.07, 0.08)	0.937		
Other	0.09 (−0.05, 0.23)	0.222		

^a^Adjusted for variables associated with MCP-1 levels in unadjusted analysis or HIV status (i.e. age, ALT levels, and HCV RNA levels) as well as assay run (*n* = 117, *R* ^2^ = 0.14)

^b^β coefficient

^c^Overall *P =* 0.467

In unadjusted linear regression analysis, HIV co-infection was also significantly associated with higher plasma eotaxin levels (log pg/mL; β: 0.17; 95 % CI: 0.07, 0.28; Table 4). In the adjusted model, HIV co-infection remained significantly associated with higher eotaxin levels (log pg/mL; adjusted β: 0.12; 95 % CI: 0.01, 0.24; Table 4).

**Table 4:** Unadjusted and adjusted models assessing the association of HIV co-infection with plasma eotaxin levels (log_10_ pg/mL) in ATAHC

	Unadjusted model		Adjusted model^a^	
	Estimated mean difference^b^ (95 % CI)	*P*	Estimated mean difference^b^ (95 % CI)	*P*
HIV co-infection				
Negative	Reference		Reference	
Positive	0.17 (0.07, 0.28)	<0.001	0.12 (0.01, 0.24)	0.038
Age, 10 years	0.08 (0.03, 0.13)	0.001	0.06 (0.00, 0.12)	0.039
Symptomatic acute HCV				
No	Reference			
Yes	0.05 (−0.05, 0.16)	0.309		
ALT level, log IU/L	0.03 (−0.07, 0.14)	0.534	−0.02 (−0.13, 0.09)	0.712
Estimated duration of infection				
<26 weeks	Reference			
≥26 weeks	−0.05 (−0.15, 0.06)	0.375		
*Interferon lambda rs12979860* genotype				
TT/CT	Reference			
CC	0.05 (−0.06, 0.15)	0.379		
HCV RNA level				
<400,000 IU/mL	Reference		Reference	
≥400,000 IU/mL	0.01 (−0.10, 0.13)	0.791	−0.02 (−0.14, 0.09)	0.689
HCV genotype^c^				
Genotype 1	Reference			
Genotype 3	−0.08 (−0.19, 0.04)	0.184		
Other	0.02 (−0.19, 0.23)	0.860		

^a^Adjusted for variables associated with eotaxin levels in unadjusted analysis or HIV status (i.e. age, ALT levels, and HCV RNA levels) as well as assay run (*n* = 117, *R* ^2^ = 0.15)

^b^ β coefficient

^c^Overall *P =* 0.372

Given that there were no females with HIV co-infection, models were not adjusted for sex. However, in a sensitivity analysis restricting the study population to males, a similar trend was observed with respect to the relationship between HIV infection and both MCP-1 (Additional file 1: Table S2) and eotaxin (Additional file 1: Table S3).

Among individuals with HIV co-infection, the mean plasma levels of MCP-1 and exotoxin among individuals with CD4 count ≥500 cells/μL were 2.12 log pg/mL [standard deviation (SD): 0.21] and 1.78 log pg/mL (SD: 0.19), respectively, which were comparable to 2.09 log pg/mL (SD: 0.18) and 1.83 log pg/mL (SD: 0.27), respectively among those with CD4 count 200–499 cells/μL (*P* = 0.627 and *P* = 0.524, respectively).

In another analysis, the association of estimated of duration of HCV infection at the time of HCV detection with plasma cytokines levels were assessed (Additional file 1: Table S4). The mean plasma levels of IL-2 was significantly higher in individuals with an estimated of duration of HCV infection ≥26 weeks (1.07 log pg/mL; SD: 0.08) compared to those with an estimated of duration of HCV infection <26 weeks (1.15 log pg/mL; SD: 0.15; *P* = 0.001).

## Discussion

This study assessed the association of plasma cytokine and chemokine levels with HIV co-infection among individuals with recent HCV infection. HIV/HCV co-infection was independently associated with higher plasma levels of two pro-inflammatory and pro-fibrogenic chemokines, MCP-1 and eotaxin. Increased plasma levels of MCP-1 and eotaxin in HIV/HCV co-infection might reflect increased hepatic expression of these cytokines and a subsequent chronic pro-inflammatory response [21, 22].

Individuals with HIV/HCV co-infection and recent HCV infection in this study had significantly higher MCP-1 plasma levels compared to those with HCV mono-infection, which is consistent with findings in the setting of chronic HCV infection [9]. Similar findings in the setting of recent HCV infection are important and suggest that elevated levels of MCP-1 in HIV/HCV co-infected individuals occur early following HCV infection. A previous study has shown that MCP-1 plasma levels were elevated in HIV infection and correlated with HIV RNA levels [23]. However, MCP-1 plasma levels in the current study were comparable between HIV co-infected individuals with and without HIV viral suppression.

C-C chemokine receptor 2 (CCR2) and its main ligand MCP-1 (CCL2) have major roles in promoting the accumulation and activation of monocyte-macrophages in the inflamed liver, as well as the activation of hepatic stellate cells (HSC) which are key drivers of fibrosis (reviewed in [24]). The role of CCR2 in promoting HSC chemotaxis and the development of hepatic fibrosis has been shown in animal models [25]. In humans, increased MCP-1 expression and CCR2-dependent macrophage infiltration in the liver [26, 27], and also higher MCP-1 plasma levels [28] have been found in the fibrotic liver. An MCP-1 gene polymorphism has been associated with increased expression of MCP-1 in the liver among individuals with chronic HCV and those with more advanced fibrosis [29]. In one longitudinal study, more rapid progression of hepatic fibrosis in HCV infection was correlated with persistent and significant elevation of MCP-1 plasma levels from acute to chronic infection [30]. Taken together, these data indicate the critical role of MCP-1 in development and progression of liver fibrosis. This existing evidence, coupled with our findings of increased MCP-1 plasma levels in HIV/HCV co-infection suggest a potential explanation for accelerated liver fibrosis progression in HIV/HCV co-infection, compared to HCV mono-infection. This hypothesis is supported by the in vitro data indicating that HIV can infect activated hepatic stellate cells (HSCs) to induce secretion of MCP-1 [31].

Knockout of CCR2 in mice results in reduced MCP-1 expression, diminished monocyte/macrophage infiltration and the development of lower levels of fibrosis following liver injury [32]. Further, data from animal models indicate that inhibition of MCP-1 reduces intrahepatic macrophage accumulation and development of steatohepatitis [21, 33]. Recently, Cenicriviric, a CCR2 and CCR5 antagonist, demonstrated good efficacy in suppressing HIV in phase IIb clinical trials [34, 35]. In addition to the antiretroviral effects, in animal models, Cenicriviric is anti-fibrotic, and reduces liver fibrosis progression [36]. One hypothesis is that the inhibition of MCP-1 might have therapeutic potential in reducing liver fibrosis progression in individuals with HIV/HCV co-infection. This hypothesis is required to be supported by further research to evaluate the potential effect of inhibiting MCP-1 in reducing liver fibrosis.

Individuals with HIV/HCV co-infection and recent HCV infection in this study also demonstrated higher plasma eotaxin levels compared to those with HCV mono-infection. Eotaxin (CCL11) is a chemokine originally known as an eosinophil-specific chemoattractant which regulates eosinophil trafficking and facilitates eosinophil migration into the tissue by activating CCR3 receptors (reviewed in [37, 38]). Up-regulation of eotaxin expression has been demonstrated in the liver of individuals with drug-induced hepatitis and is accompanied by liver infiltration of eosinophils [39]. In the setting of HCV infection, increased plasma levels of eotaxin have been demonstrated in individuals with chronic HCV infection compared with healthy controls [40], while development of persistent HCV has also been found to be associated with higher plasma levels of eotaxin during acute infection [41]. Moreover, eotaxin has a role in hepatic fibrogenesis. Higher plasma eotaxin levels have been identified among individuals with liver cirrhosis, with higher eotaxin levels associated with increasing stage of fibrosis, and hepatic necro-inflammation and fibrosis by liver histology [42]. The observation that higher eotaxin plasma levels are observed in those with HIV/HCV co-infection suggest that, similar to MCP-1, eotaxin might have a role in explaining accelerated liver fibrosis in individuals with HIV/HCV co-infection.

This study had several limitations. First, this dataset was cross-sectional, then it was not possible to measure longitudinal levels of cytokines during acute HCV infection. Further, cytokine and chemokine concentrations were measured from plasma samples, so it is possible that the levels in the blood might not reflect hepatic levels. However, it has been demonstrated that intrahepatic and peripheral sources of MCP-1 are known to both contribute to elevated serum MCP-1 concentrations [22]. Data on liver fibrosis levels was not available in our participants. Further studies are needed to investigate the role of high MCP-1 and eotaxin levels in liver fibrosis progression among HIV/HCV co-infected individuals. Lastly, multiple comparison testing might inflate type 1 error in data analysis. To account for this concern, a moderately conservative significance level (*alpha* = 0.01) was used. We also conducted a sensitivity analysis, using Bonferroni correction for multiple comparisons (*alpha* = 0.05/27 = 0.002) but the findings were similar.

## Conclusion

In conclusion, this study has demonstrated that compared to those without HIV infection, following acute HCV infection among people with HIV-infection, increased levels of the pro-fibrogenic chemokines, MCP-1 and eotaxin were observed. These findings could suggest a potential pathway possibly linking HIV infection with liver fibrogenesis among people with HIV/HCV infection. These findings may have also potential implications for therapeutic interventions to prevent liver fibrosis in people with HIV/HCV co-infection.

## Abbreviations

ALT, alanine aminotransferase; ATAHC, Australian trial in acute hepatitis C; HCV, hepatitis C virus; IFN, interferon-gamma; IL, interleukin; IP-10, interferon-gamma inducible protein-10; IQR, inter-quartile range; MCP, monocyte chemotactic protein; MIP, macrophage inflammatory protein; RANTES, regulated upon activation normal T-cell expressed and presumably secreted; sCD40L, soluble CD40 ligand; TNF, tumor necrosis factor; TRAIL, TNF-related apoptosis-inducing ligand.

## Additional file

Additional file 1:
**Table S1.** The lowest level of detection for measurement of plasma cytokine and chemokine levels. **Table S2.** Unadjusted and adjusted models assessing the association of HIV co-infection with plasma MCP-1 levels among males in ATAHC (*n*=88). **Table S3.** Unadjusted and adjusted models assessing the association of HIV co-infection with plasma eotaxin level among males in ATAHC (*n*=88). **Table S4.** Plasma cytokine and chemokine levels among ATAHC participants with detectable HCV RNA at the time of acute HCV detection, stratified by estimated duration of HCV infection at the time of HCV detection. (DOCX 32 kb)
