# Supplementary material for: HIV infection is associated with higher levels of monocyte chemoattractant protein-1 and eotaxin among people with recent hepatitis C virus infection
Source: BMC Infect Dis. 2016 Jun 1;16:241. doi: 10.1186/s12879-016-1567-2 (PMC4888248; doi:10.1186/s12879-016-1567-2)
Supplement: Additional file 1: — Table S1. The lowest level of detection for measurement of plasma cytokine and chemokine levels. Table S2. Unadjusted and adjusted models assessing the association of HIV co-infection with plasma MCP-1 levels among males in ATAHC (n=88). Table S3. Unadjusted and adjusted models assessing the association of HIV co-infection with plasma eotaxin level among males in ATAHC (n=88). Table S4. Plasma cytokine and chemokine levels among ATAHC participants with detectable HCV RNA at the time of acute HCV detection, stratified by estimated duration of HCV infection at the time of HCV detection. (DOCX 32 kb) [file 12879_2016_1567_MOESM1_ESM.docx]

**S1 Table . The lowest level of detection for measurement of plasma cytokine and chemokine levels**

| Cytokine | Lowest level of detection (pg/mL) |
| --- | --- |
| IL-1 beta | 0.274 |
| IL-2 | 0.479 |
| IL-4 | 5.113 |
| IL-6 | 1.173 |
| IL-8 | 1.346 |
| IL-10 | 2.108 |
| IL-17A | 1.681 |
| IL-17F | 9.629 |
| IL-18 | 0.741 |
| IL-21 | 15.798 |
| IL-22 | 4.682 |
| IL-23 | 29.334 |
| IL-25 | 1.233 |
| IL-31 | 12.631 |
| IL-33 | 3.387 |
| IFN-gamma | 46.458 |
| IFN-gamma2 | 41.454 |
| TNF-alpha | 0.283 |
| TNF-beta | 1.154 |
| TRAIL | 2.570 |
| sCD40L | 2.551 |
| CXCL10 (IP10) | 2.376 |
| CCL2 (MCP-1) | 1.037 |
| CCL3 (MIP1a) | 1.151 |
| CCL4 (MIP1b) | 1.326 |
| CCL5 (RANTES) | 5.151 |
| CCL11 (eotaxin) | 1.774 |

**S2 Table. Unadjusted and adjusted models assessing the association of HIV co-infection with plasma MCP-1 levels among males in ATAHC (n=88)**

|  | Unadjusted model | | Adjusted model^*^ | |
| --- | --- | --- | --- | --- |
|  | Estimated Mean difference^**^ (95% CI) | *P* | Estimated Mean difference^**^ (95% CI) | *P* |
| **HIV co-infection** |  |  |  |  |
| Negative | Reference |  | Reference |  |
| Positive | 0.08 (0.00, 0.16) | 0.040 | 0.07 (-0.02, 0.16) | 0.130 |
| **Age, 10 years** | 0.03 (-0.01, 0.07) | 0.095 | 0.02 (-0.03, 0.06) | 0.498 |
| **Symptomatic acute HCV** |  |  |  |  |
| No | Reference |  |  |  |
| Yes | 0.05 (-0.03, 0.13) | 0.199 |  |  |
| **ALT level, log IU/L** | 0.04 (-0.05, 0.13) | 0.384 | 0.02 (-0.07, 0.12) | 0.636 |
| **Estimated duration of infection** |  |  |  |  |
| <26 weeks | Reference |  |  |  |
| ≥26 weeks | -0.00 (-0.09, 0.08) | 0.944 |  |  |
| ***Interferon lambda rs12979860* genotype** |  |  |  |  |
| TT/CT | Reference |  |  |  |
| CC | 0.00 (-0.09, 0.08) | 0.959 |  |  |
| **HCV RNA level** |  |  |  |  |
| <400,000 IU/mL | Reference |  | Reference |  |
| ≥400,000 IU/mL | 0.02 (-0.06, 0.11) | 0.594 | 0.00 (-0.09, 0.10) | 0.952 |
| **HCV genotype**^†^ |  |  |  |  |
| Genotype 1 | Reference |  |  |  |
| Genotype 3 | 0.01 (-0.08, 0.10) | 0.837 |  |  |
| Other | 0.07 (-0.10, 0.23) | 0.410 |  |  |

*Adjusted for variables associated with MCP-1 levels in unadjusted analysis or HIV status as well as assay run (n=88, R^2^=0.10)

**β coefficient

†Overall *P=*0.710

**S3 Table. Unadjusted and adjusted models assessing the association of HIV co-infection with plasma eotaxin level among males in ATAHC (n=88)**

|  | Unadjusted model | | Adjusted model^*^ | |
| --- | --- | --- | --- | --- |
|  | Estimated Mean difference^**^ (95% CI) | *P* | Estimated Mean difference^**^ (95% CI) | *P* |
| **HIV co-infection** |  |  |  |  |
| Negative | Reference |  | Reference |  |
| Positive | 0.14 (0.04, 0.23) | 0.005 | 0.10 (-0.00, 0.20) | 0.055 |
| **Age, 10 years** | 0.07 (0.02, 0.12) | 0.003 | 0.05 (-0.00, 0.10) | 0.055 |
| **Symptomatic acute HCV** |  |  |  |  |
| No | Reference |  |  |  |
| Yes | 0.05 (-0.05, 0.15) | 0.294 |  |  |
| **ALT level, log IU/L** | -0.06 (-0.16, 0.05) | 0.308 | -0.07 (-0.18, 0.04) | 0.192 |
| **Estimated duration of infection** |  |  |  |  |
| <26 weeks | Reference |  |  |  |
| ≥26 weeks | -0.02 (-0.13, 0.08) | 0.669 |  |  |
| ***Interferon lambda rs12979860* genotype** |  |  |  |  |
| TT/CT | Reference |  |  |  |
| CC | 0.01 (-0.09, 0.11) | 0.785 |  |  |
| **HCV RNA level** |  |  |  |  |
| <400,000 IU/mL | Reference |  | Reference |  |
| ≥400,000 IU/mL | -0.01 (-0.11, 0.09) | 0.875 | -0.02 (-0.13, 0.09) | 0.730 |
| **HCV genotype**^†^ |  |  |  |  |
| Genotype 1 | Reference |  |  |  |
| Genotype 3 | -0.06 (-0.16, 0.05) | 0.295 |  |  |
| Other | 0.01 (-0.19, 0.21) | 0.931 |  |  |

*Adjusted for variables associated with eotaxin levels in unadjusted analysis or HIV status as well as assay run (n=88, R^2^=0.20)

**β coefficient

†Overall *P=* 0.551

**S4 Table. Plasma cytokine and chemokine levels among ATAHC participants with detectable HCV RNA at the time of acute HCV detection, stratified by estimated duration of HCV infection at the time of HCV detection**

| Cytokine | <26 weeks  *(n=69)* | ≥26 weeks  *(n=48)* | *P* |
| --- | --- | --- | --- |
| IL-1 beta^*^ | 0.63 (0.89) | 0.77 (0.75) | 0.373 |
| IL-2^*^ | 1.07 (0.08) | 1.15 (0.15) | 0.001 |
| IL-4^*^ | 2.12 (0.23) | 2.19 (0.30) | 0.162 |
| IL-6^*^ | 1.55 (0.62) | 1.67 (0.66) | 0.319 |
| IL-8^*^ | 1.70 (0.47) | 1.73 (0.58) | 0.756 |
| IL-10^*^ | 2.04 (0.67) | 2.13 (0.72) | 0.505 |
| IL-17A^*^ | 1.91 (0.63) | 2.11 (0.55) | 0.076 |
| IL-17F^*^ | 2.23 (0.53) | 2.34 (0.51) | 0.244 |
| IL-18^*^ | 2.47 (0.26) | 2.47 (0.30) | 0.944 |
| IL-21^*^ | 3.12 (0.51) | 3.09 (0.61) | 0.763 |
| IL-22^*^ | 1.69 (0.66) | 1.86 (0.56) | 0.131 |
| IL-23^**^ | 20 (29) | 10 (21) | 0.299 |
| IL-25^*^ | 1.37 (0.80) | 1.46 (0.82) | 0. 573 |
| IL-31^*^ | 2.51 (0.56) | 2.64 (0.53) | 0.187 |
| IL-33^*^ | 3.42 (0.26) | 3.48 (0.29) | 0.303 |
| IFN-gamma^*^ | 2.63 (0.41) | 2.66 (0.31) | 0.715 |
| IFN-gamma2^**^ | 58 (85) | 37 (77) | 0.258 |
| TNF-alpha^*^ | 1.30 (0.48) | 1.27 (0.44) | 0.740 |
| TNF-beta^**^ | 68 (98) | 43 (90) | 0.042 |
| TRAIL^*^ | 2.08 (0.18) | 2.10 (0.20) | 0.430 |
| sCD40L^*^ | 2.88 (0.27) | 2.90 (0.24) | 0.651 |
| CXCL10 (IP-10)^*^ | 3.04 (0.26) | 3.01 (0.25) | 0.540 |
| CCL2 (MCP-1)^*^ | 2.05 (0.20) | 2.00 (0.18) | 0.196 |
| CCL3 (MIP-1a)^**^ | 29 (42) | 23 (48) | 0.528 |
| CCL4 (MIP-1b)^*^ | 2.64 (0.26) | 2.65 (0.30) | 0.937 |
| CCL5 (RANTES)^*^ | 3.97 (1.49) | 3.65 (1.79) | 0.291 |
| CCL11 (eotaxin)^*^ | 1.71 (0.23) | 1.66 (0.34) | 0.407 |

* Presented as mean plasma level (standard deviation); log_10_ pg/mL

** Presented as the number with undetectable plasma level (%)
